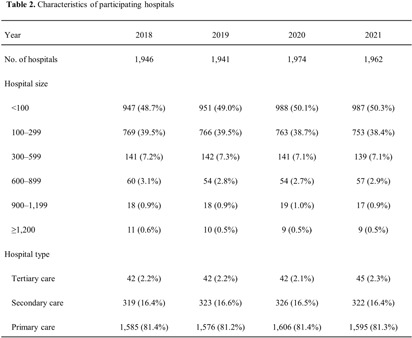# Nationwide analysis of antimicrobial prescription in Korean hospitals between 2018 and 2021: The 2023 KONAS report

**DOI:** 10.1017/ash.2024.150

**Published:** 2024-09-16

**Authors:** Yong Chan Kim, I Ji Yun, Hyo Jun Park, Jungmi Chae, Hyung-sook Kim, Song Mi Moon, Eunjeong Heo, Se Yoon Park, Dong Min Seo, Ha-Jin Chun, Myung Jin Lee, Kyungmin Huh, Su Jin Jeong, Jun Yong Choi, Bongyoung Kim

**Affiliations:** Yongin Severance Hospital; Yonsei University College of Medicine; SNUBH; Ajou University Hospital Pharmacy; Hanyang University

## Abstract

**Background:** Data on antimicrobial use at the national level is crucial to establish domestic antimicrobial stewardship policies and enable medical institutions to benchmark against each other. This study aimed to analyze antimicrobial use in Korean hospitals. **Methods:** We investigated the antimicrobials prescribed in Korean hospitals between 2018 and 2021, using data from the Health Insurance Review and Assessment. Primary care hospitals (PCHs), secondary care hospitals (SCHs), and tertiary care hospitals (TCHs) were included in this analysis. Antimicrobials were categorized according to the Korea National Antimicrobial Use Analysis System (KONAS) classification, which is suitable for measuring antimicrobial use in Korean hospitals. **Results:** Out of more than 1,900 hospitals, PCHs and TCHs represented the largest and lowest percentage of hospitals, respectively. The most frequently prescribed antimicrobial in 2021 was piperacillin/β-lactamase inhibitor (9.3%) in TCHs, ceftriaxone (11.0%) in SCHs, and cefazedone (18.9%) in PCHs. Between 2018 and 2021, the most used antimicrobial class according to the KONAS classification was ‘broad-spectrum antibacterial agents predominantly used for community-acquired infections’ in TCHs and SCHs, and 'narrow spectrum beta-lactam agents' in PCH. Total consumption of antimicrobials has decreased from 951.7 to 929.9 days of therapy (DOT)/1,000 patient-days in TCHs and from 817.8 to 752.2 DOT/1,000 patient-days in SCHs during study period, but not in PCHs (from 504.3 to 527.2 DOT/1,000 patient-days). Moreover, in 2021, while use of reserve antimicrobials has decreased from 13.6 to 10.7 DOT/1,000 patient-days in TCHs and from 4.6 to 3.3 DOT/1,000 patient-days in SCHs, it has increased from 0.7 to 0.8 DOT/1,000 patient-days in PCHs. **Conclusion:** This study confirms that antimicrobial use differs by hospital type in Korea. Recent increases of use of antimicrobials, including reserve antimicrobials, in PCHs reflect the challenges that must be addressed.

**Figure:**
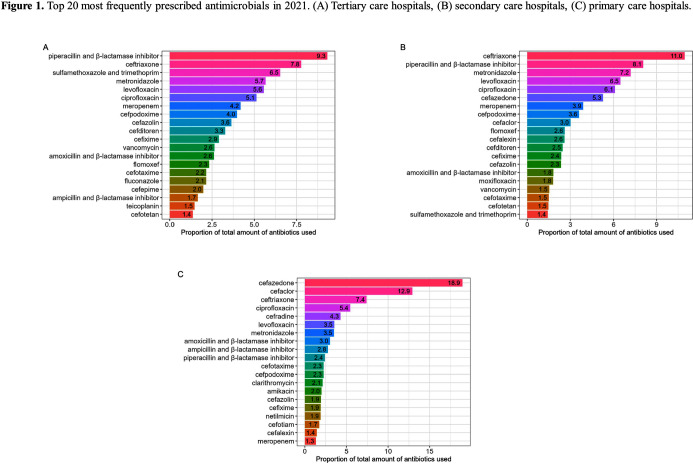

**Figure:**
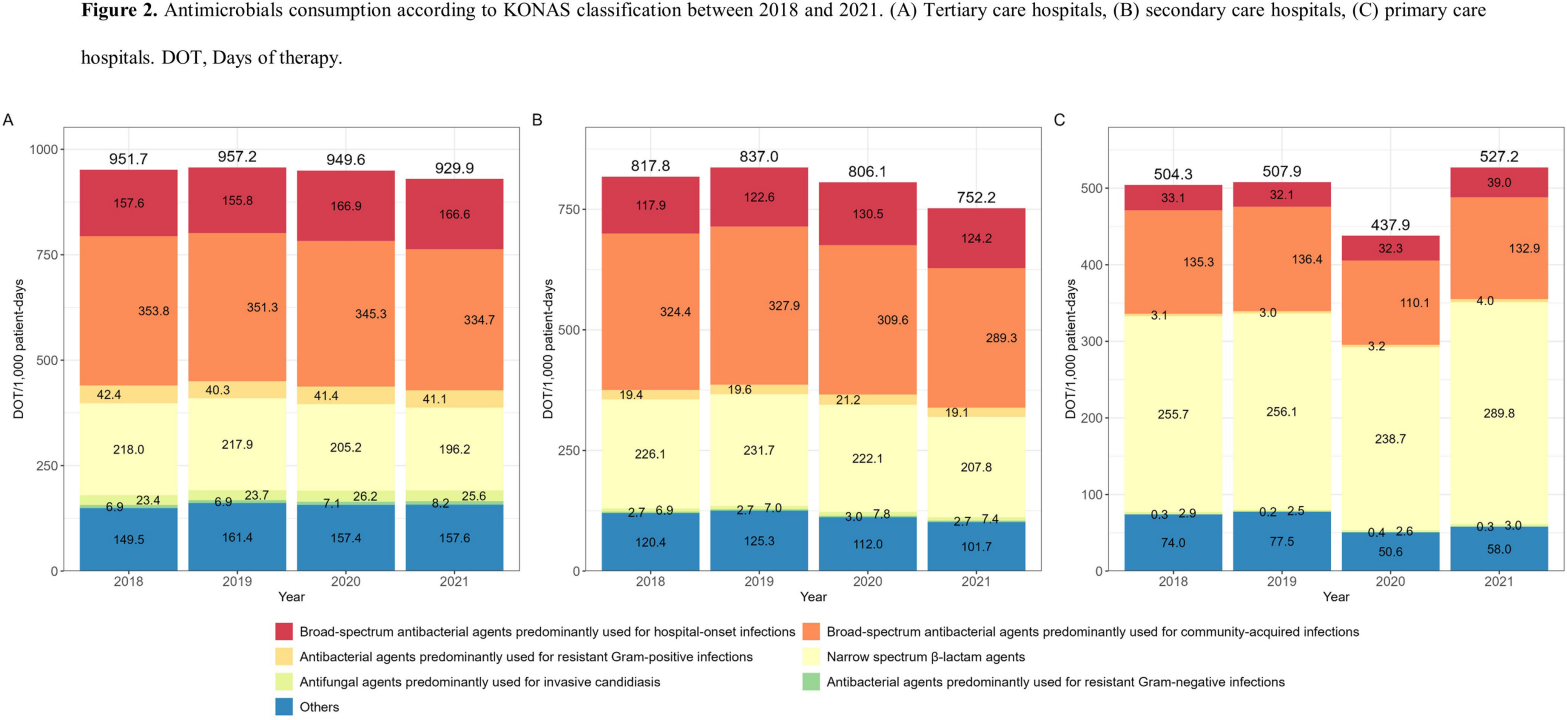

**Figure:**
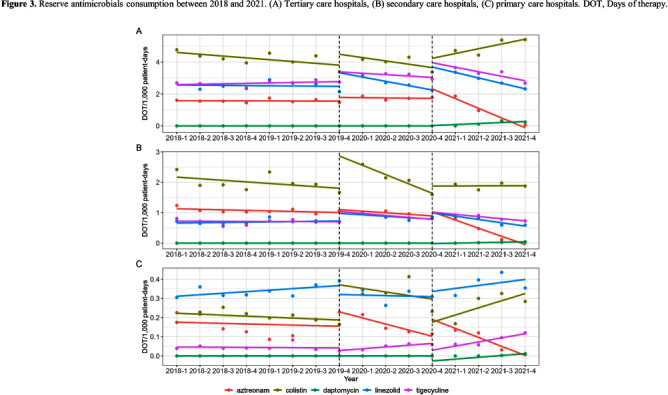

**Figure:**
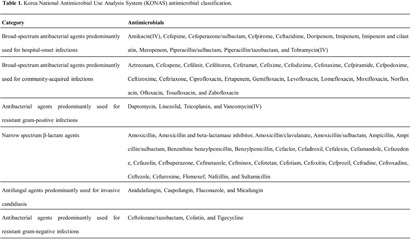

**Figure:**